# PPIs Are Not Responsible for Elevating Cardiovascular Risk in Patients on Clopidogrel—A Systematic Review and Meta-Analysis

**DOI:** 10.3389/fphys.2018.01550

**Published:** 2018-11-19

**Authors:** Alexandra Demcsák, Tamás Lantos, Emese Réka Bálint, Petra Hartmann, Áron Vincze, Judit Bajor, László Czopf, Hussain Alizadeh, Zoltán Gyöngyi, Katalin Márta, Alexandra Mikó, Zsolt Szakács, Dániel Pécsi, Péter Hegyi, Imre László Szabó

**Affiliations:** ^1^Department of Pediatrics and Pediatric Health Centre, University of Szeged, Szeged, Hungary; ^2^Department of Medical Physics and Informatics, University of Szeged, Szeged, Hungary; ^3^First Department of Internal Medicine, University of Szeged, Szeged, Hungary; ^4^Institute of Surgical Research, University of Szeged, Szeged, Hungary; ^5^Division of Gastroenterology, First Department of Internal Medicine, University of Pécs Medical School, Pécs, Hungary; ^6^Division of Cardiology, First Department of Internal Medicine, University of Pécs Medical School, Pécs, Hungary; ^7^Division of Hematology, First Department of Internal Medicine, University of Pécs Medical School, Pécs, Hungary; ^8^Department of Public Health Medicine, University of Pécs Medical School, Pécs, Hungary; ^9^Institute for Translational Medicine, University of Pécs Medical School, Pécs, Hungary; ^10^János Szentágothai Research Centre, University of Pécs, Pécs, Hungary; ^11^Momentum Translational Gastroenterology Research Group, Hungarian Academy of Sciences, University of Szeged, Szeged, Hungary

**Keywords:** proton pump inhibitors, clopidogrel, cardiovascular risk, drug interaction, cytochrome P450, meta-analysis

## Abstract

**Background:** Clopidogrel and proton pump inhibitors (PPIs) are metabolized by cytochrome P450 enzymes. Contradictory results have been reported on possible complications of simultaneous PPI and clopidogrel use. Our aim was to investigate the clinical relevance of this debate with a systematic review and meta-analysis.

**Methods:** The PubMed, Embase, and Cochrane Central Register of Controlled Trials electronic databases were searched for human studies [randomized controlled trials (RCTs) and observational studies] using the PICO format (P: patients on clopidogrel; I: patients treated with PPI; C: patients without PPI treatment; O: cardiovascular risk). We screened eligible studies from 2009 to 2016. After study exclusions, we extracted data from 27 articles for three outcomes: major adverse cardiac event (MACE), myocardial infarction (MI) and cardiovascular (CV) death. The meta-analysis was registered on PROSPERO (CRD42017054316).

**Results:** Data were extracted on 156,823 patients from the 27 trials included (MACE: 23, CV death: 10, MI: 14). The risks of MACE (RR = 1.22, 95% CI = 1.06–1.396, *p* = 0.004) and MI (RR = 1.43, 95% CI = 1.24–1.66, *p* < 0.001) were significantly higher in the PPI plus clopidogrel group. However, subgroup analysis demonstrated that this significance disappeared in RCTs (RR = 0.99, 95% CI = 0.76–1.28, *p* = 0.93) in the MACE outcome group. There was no effect of combined PPI and clopidogrel therapy on CV death outcome (RR = 1.21, 95% CI = 0.97–1.50, *p* = 0.09).

**Conclusion:** Concomitant use of PPIs and clopidogrel has been proved not to be associated with elevated cardiovascular risks according to RCTs. Based on our results, no restrictions should be applied whenever PPIs and clopidogrel are administered simultaneously.

## Introduction

The literature consists of contradictory findings on the concomitant usage of clopidogrel and proton pump inhibitors (PPIs). A combination of antiplatelet drugs is used for the treatment of acute coronary syndrome (i.e., aspirin and thienopyridines) and for the secondary prevention of further cardiovascular (CV) events (Yusuf et al., 2001). It is well-documented that dual antiplatelet therapy is followed by possible side-effects, such as higher risk for gastrointestinal (GI) bleeding increasing both mortality and ischaemic complications (Nikolsky et al., 2009; Disney et al., 2011). To reduce the risk of GI bleeding in patients with risk factors, PPIs are strongly recommended by the American College of Cardiology, the American College of Gastroenterology, and the American Heart Association (Bhatt et al., 2008; Abraham et al., 2010; Disney et al., 2011). *In vitro* findings suggested that PPIs reduce the antiplatelet effect of clopidogrel (Gilard et al., 2008), followed by several clinical studies with contradictory outcomes (Pezalla et al., 2008; Ho et al., 2009; Juurlink et al., 2009; O'Donoghue et al., 2009; Rassen et al., 2009; Bhatt et al., 2010; Charlot et al., 2010; Gupta et al., 2010; Hudzik et al., 2010; Kreutz et al., 2010; Ray et al., 2010; van Boxel et al., 2010; Zairis et al., 2010; Burkard et al., 2012; Mo et al., 2015; Sherwood et al., 2015). A higher risk for CV outcomes was found in several studies, systematic reviews and meta-analyses in patients with clopidogrel on PPI therapy. Generally, whenever observational studies were included, a positive association was described. On the other hand, whenever propensity-matched groups were compared the difference between the groups disappeared (Rassen et al., 2009; Kwok and Loke, 2010; Valkhoff et al., 2011; Chen et al., 2012; Mo et al., 2015). Therefore, it is clear that a precise investigation is crucial to understanding the potential CV risk of co-administration of clopidogrel and PPIs.

## Materials and methods

### Literature search

**Figure 1:**
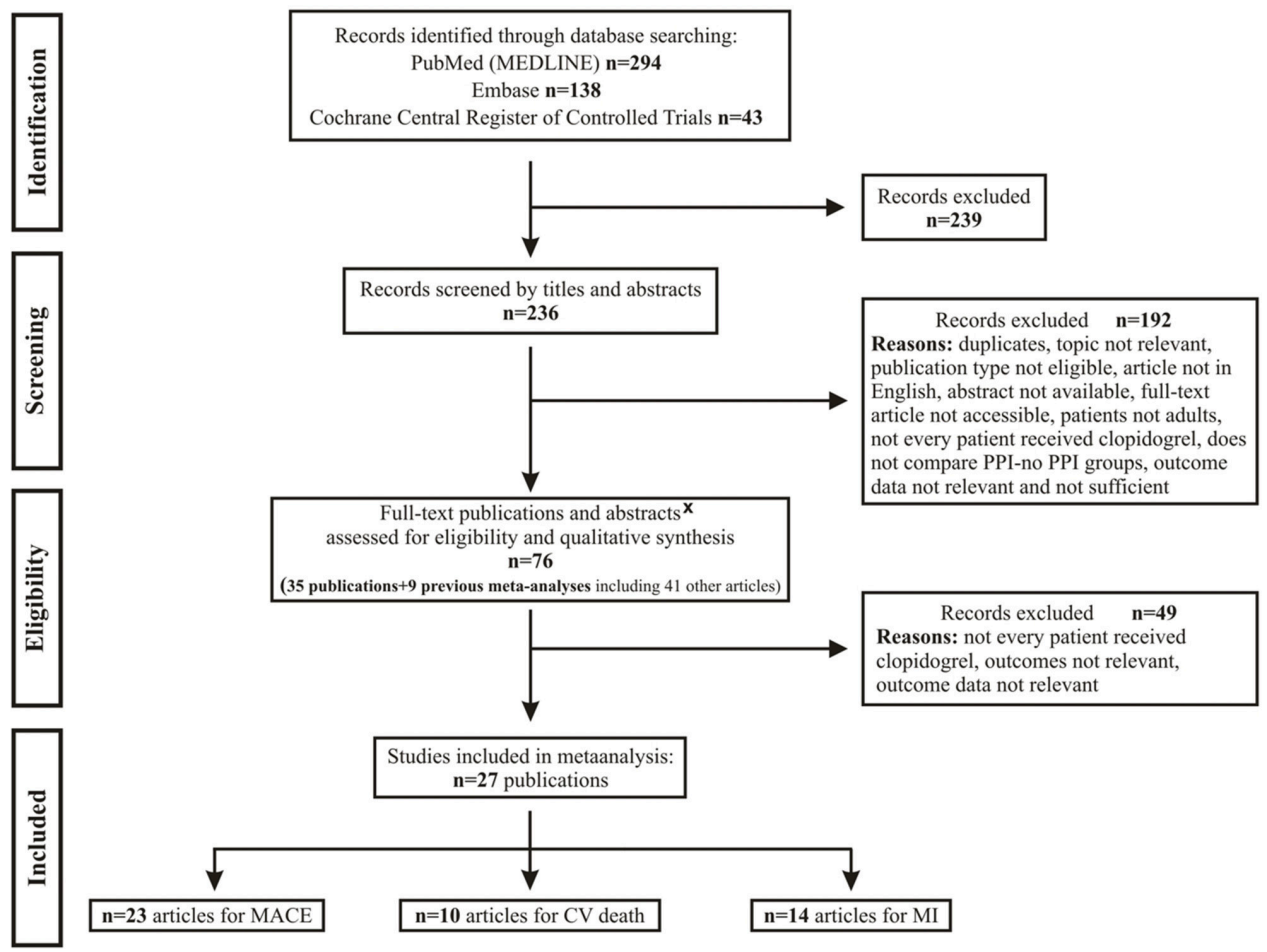
Flowchart for study selection and inclusion. CV, cardiovascular; MACE, major adverse cardiac event; MI, myocardial infarction; PPI, proton pump inhibitor; x, full articles were not available by any suitable sources.

A systematic review of studies was performed in accordance with the Preferred Reporting Items for Systematic Reviews and Meta-analysis (PRISMA) Statement (Moher et al., 2015). After developing our clinical question and translating it into a well-defined systematic review question based on the PICO format (Patients, Interventions, Comparators and Outcomes), a manual search of medical databases, including PubMed (MEDLINE), Embase, and the Cochrane Central Register of Controlled Trials, was performed for human observations using the following PICO format: P: patients on clopidogrel; I: patients treated with PPI; C: patients without PPI treatment; O: cardiovascular risk. Two independent investigators (AD and ERB) separately screened the titles and abstracts for eligible studies published from inception to 30 December 2016. The flowchart for this process is shown in Figure 1. After searching the international prospective register for systematic reviews (PROSPERO) for ongoing or completed meta-analyses on the examined effects of PPIs, we registered our present meta-analysis on PROSPERO under No. CRD42017054316.

### Study selection

Inclusion criteria: (1) randomized or observational studies (cohort and case-control studies) carried out either in a retro- or prospective manner; (2) only adult patients (over 18 years); (3) patients receiving clopidogrel treatment; (4) should compare PPI takers (omeprazole, pantoprazole, esomeprazole, lansoprazole and/or rabeprazole; all doses) and non-PPI takers; (5) we only involved studies that stated exact patient number in the preferred groups (total number of patients, patients who received clopidogrel and PPI, outcome number); (6) human studies; (7) studies should show data for either one or more of the following outcomes: (1) major adverse cardiac event (MACE): composite of cardiac and non-cardiac death, non-fatal myocardial infarction, target vessel failure; (2) myocardial infarction (MI): myocardial infarction or new, definitive major coronarographic defect; (3) CV death: only CV death. Studies published in English were selected. Duplicates were eliminated from the analysis manually. Disagreements were resolved by consulting a small committee of three researchers (PeH, JB, and ÁV).

### Data extraction

Numeric and texted data were extracted from the eligible articles as follows: author, publication year, study type, study endpoints, number of patients in the study, in PPI and in non-PPI treatment groups, and number of patients who received clopidogrel. We also collected the specified generic name of the PPI and patient number if indicated. For study characteristics we collected numeric and texted data as follows: country/region, mean follow up, number of male patients, mean age and mean body mass index, other medications (angiotensin-converting enzyme inhibitor, angiotensin receptor blocker, statin), cardio- and cerebrovascular history (MI, percutaneous coronary intervention, stroke) and CV risk factors (hypertension, diabetes mellitus, dyslipidaemia, smoking) in the non-PPI and PPI groups (Supplementary Tables 1A–D).

### Risk of bias

The Newcastle–Ottawa quality assessment scale (Wells et al., 2013) has been edited to our study design, and was used to assess the quality of observational studies and *post-hoc* analyses of randomized controlled trials (RCTs) (For further details see Supplementary Material, Supplementary Figure 7B). We used the Cochrane risk of bias tool (Higgins et al., 2011) for quality assessment of RCTs (Supplementary Figure 7A).

### Statistical analysis

We calculated risk ratio/relative risk (RR) and 95% confidence interval (CI) for CV events (MACE, MI and CV death). As secondary analyses, we calculated pooled hazard ratios and 95% CI for the adjusted events for all three major outcomes (Supplementary Figures 4–6). Between-study heterogeneity was tested with the *I*^2^ statistic, where *I*^2^ is the proportion of total variation attributable to between-study variability. *I*^2^ heterogeneity was interpreted according to the Cochrane Handbook for Systematic Reviews and Interventions recommendation: 0–40%: might not be important; 30–60%: may represent moderate heterogeneity; 50–90%: may represent substantial heterogeneity; 75–100%: considerable heterogeneity (Higgins and Green, 2011). Fixed or random effects models were used for comparison between the two groups (clopidogrel alone or clopidogrel plus PPI), based on the degree of heterogeneity, or based on methodological factors such as difference between study designs or applied PPIs, not homogeneous patient population etc. We estimated the effect of follow up and age on the risk of the three major outcomes by performing random effects meta-regression expressed as standard error and 95% CI. *P*-values of < 0.05 for relative risks and standard errors, and *p*-values of < 0.10 for heterogeneity were considered as indicators of significance. We estimated publication bias through a visual inspection of funnel plots (Figures 5A–C). The statistical analysis was performed by a trained biostatistician (TL). All analyses were performed with the Review Manager (RevMan) software, Version 5.3 (Copenhagen: The Nordic Cochrane Centre, The Cochrane Collaboration, 2014).

## Results

### Study selection

Two hundred and thirty-six articles were identified in the preliminary search. One hundred and ninety-three studies were excluded (Figure 1). Seventy-six publications (25 full texts, 10 abstracts, and 41 articles from previous meta-analyses) were assessed for eligibility and qualitative synthesis. Forty-seven of them were excluded due to insufficient data on study groups and another two for statistical reasons (the event rate was zero). A total of 27 studies (Rassen et al., 2009; Bhatt et al., 2010; Cai et al., 2010; Charlot et al., 2010; Evanchan et al., 2010; Gupta et al., 2010; Hudzik et al., 2010; Kreutz et al., 2010; Ray et al., 2010; Stockl et al., 2010; van Boxel et al., 2010; Hsu et al., 2011; Ren et al., 2011; Rossini et al., 2011; Simon et al., 2011; Burkard et al., 2012; Chitose et al., 2012; Goodman et al., 2012; Ng et al., 2012; Yano et al., 2012; Hokimoto et al., 2014; Shih et al., 2014; Zou et al., 2014; Weisz et al., 2015; Ayub et al., 2016; Gargiulo et al., 2016) were selected for quantitative analyses. The researchers and committee involved in the selection (5 investigators) were in total agreement on all the inclusions and exclusions.

### Study characteristics

Altogether, we found data for MACE in 23 publications (O'Donoghue et al., 2009; Bhatt et al., 2010; Cai et al., 2010; Charlot et al., 2010; Gupta et al., 2010; Hudzik et al., 2010; Kreutz et al., 2010; Ray et al., 2010; van Boxel et al., 2010; Hsu et al., 2011; Ren et al., 2011; Rossini et al., 2011; Simon et al., 2011; Burkard et al., 2012; Chitose et al., 2012; Goodman et al., 2012; Ng et al., 2012; Yano et al., 2012; Hokimoto et al., 2014; Zou et al., 2014; Weisz et al., 2015; Ayub et al., 2016; Gargiulo et al., 2016), for CV death in 10 (Rassen et al., 2009; Bhatt et al., 2010; Gupta et al., 2010; Simon et al., 2011; Burkard et al., 2012; Chitose et al., 2012; Goodman et al., 2012; Hokimoto et al., 2014; Zou et al., 2014; Weisz et al., 2015; Gargiulo et al., 2016) and for MI in 14 (Rassen et al., 2009; Bhatt et al., 2010; Evanchan et al., 2010; Hudzik et al., 2010; Stockl et al., 2010; van Boxel et al., 2010; Simon et al., 2011; Burkard et al., 2012; Chitose et al., 2012; Goodman et al., 2012; Shih et al., 2014; Zou et al., 2014; Weisz et al., 2015; Gargiulo et al., 2016). Seventeen of them were observational studies, 16 were cohorts (Rassen et al., 2009; Charlot et al., 2010; Evanchan et al., 2010; Gupta et al., 2010; Kreutz et al., 2010; Ray et al., 2010; Stockl et al., 2010; van Boxel et al., 2010; Rossini et al., 2011; Simon et al., 2011; Chitose et al., 2012; Hokimoto et al., 2014; Shih et al., 2014; Zou et al., 2014; Weisz et al., 2015; Ayub et al., 2016), and one was a case-control study (Hudzik et al., 2010). Data from 10 RCTs (O'Donoghue et al., 2009; Bhatt et al., 2010; Cai et al., 2010; Hsu et al., 2011; Ren et al., 2011; Burkard et al., 2012; Goodman et al., 2012; Ng et al., 2012; Yano et al., 2012; Gargiulo et al., 2016) were also collected. As *post-hoc* analyses of RCTs, in four studies (O'Donoghue et al., 2009; Burkard et al., 2012; Goodman et al., 2012; Gargiulo et al., 2016) the populations and outcome of our interest (clopidogrel plus PPI vs. clopidogrel plus non-PPI treatment) were not randomized, therefore, their data were included in the statistical analyses of observational studies. The method and the study selection are shown in Figure 1. All the studies included were published between 2009 and 2016. The characteristics of the studies involved in the meta-analysis are summarized in Table 1 according to the major outcome groups, and in Supplementary Tables 1A–D.

**Table 1:** Study characteristics.

**References, year**	**Study type**	**Number of patients**	**PPI** **(generic name)**	**PPI** **(number of patients)**	**Event number:** **MACE** **(PPI group)**	**Event number:****CV death****(PPI group)**	**Event number: MI****(PPI group)**
Ng et al., 2012	RCT	311	Esomeprazole	163	7	
Yano et al., 2012	RCT	130	Omeprazole	65	8	
Hsu et al., 2011	RCT	42	Esomeprazole	21	4	
Ren et al., 2011	RCT	172	Omeprazole	86	22	
Bhatt et al., 2010	RCT	3,761	Omeprazole	1,876	55	5	14
Cai et al., 2010	RCT	60	Omeprazole Pantoprazole	40	10	
Gargiulo et al., 2016	RCT (*post-hoc* analysis)	1,970	Pantoprazole Lansoprazole Omeprazole, esomeprazole, rabeprazole	738 56 671 11	85	29	41
Burkard et al., 2012	RCT (*post-hoc* analysis)	801	Esomeprazole Pantoprazole Omeprazole	109 55 27 19	33	10	25
Goodman et al., 2012	RCT (*post-hoc* analysis)	9,276	Omeprazole Pantoprazole Esomeprazole Lansoprazole Rabeprazole	3,255 1,592 973 387 251 51	398	180	245
O'Donoghue et al., 2009	RCT (*post-hoc* analysis)	13,608	Omeprazole Pantoprazole Lansoprazole Esomeprazole	4,529 1,675 1,844 441 613	255	
Ayub et al., 2016	Observational cohort	740	Omeprazole Esomeprazole Pantoprazole	332 40 81	30 6 10	
Weisz et al., 2015	Observational cohort	8,581	NS	2,162	238	58	100
Hokimoto et al., 2014	Observational cohort	174	Rabeprazole	50	5	
Shih et al., 2014	Observational cohort	2,703	NS	1,351			12
Zou et al., 2014	Observational cohort	7,653	Omeprazole Pantoprazole Esomeprazole	6,188 5,587 407 194	860	223	132
Chitose et al., 2012	Observational cohort	630	NS	187	7	4	1
Rossini et al., 2011	Observational cohort	1,328	Lansoprazole Pantoprazole Omeprazole	1,158 853 178 125	87	
Simon et al., 2011	Observational cohort	2,353	Omeprazole Esomeprazole Pantoprazole Lansoprazole	1,453 993 311 99 46	43 20 12 1	94	24
Charlot et al., 2010	Observational cohort	24,702	NS	6,753	1058	
Evanchan et al., 2010	Observational cohort	5,794	Esomeprazole Lansoprazole Omeprazole Pantoprazole	1,369 749 36 163 693			356
Gupta et al., 2010	Observational cohort	315	Rabeprazole, omeprazole, lansoprazole	72	40	14
Hudzik et al., 2010	Observational case-control	38	Omeprazole	18	10		6
Kreutz et al., 2010	Observational cohort	16,690	Omeprazole Pantoprazole Lansoprazole Esomeprazole	6,828 2,307 1,653 785 3257	1710	
Ray et al., 2010	Observational cohort	16,221	Omeprazole Pantoprazole Lansoprazole, rabeprazole, esomeprazole	7,226 683 4,708	461	
Stockl et al., 2010	Observational cohort	2,066	Pantoprazole Rabeprazole Omeprazole Lansoprazole Esomeprazole	1,033 659 159 86 83 46			133
van Boxel et al., 2010	Observational cohort	18,139	Omeprazole Pantoprazole Esomeprazole Rabeprazole Lansoprazole	5,734 1,826 2,618 1,092 133	754		84
Rassen et al., 2009	Observational cohort	18,565	Omeprazole, rabeprazole, esomeprazole, lansoprazole, pantoprazole	3,996		61	238

*CV, cardiovascular; MACE, major adverse cardiac event; MI, myocardial infarction; NS, not shown/not specified; PPI, proton pump inhibitor; RCT, randomized controlled trial*.

The number of patients involved was 156,823. A total of 63,756 received PPI plus clopidogrel treatment (ranging from 18 to 6,843), and 99,910 (ranging from 20 to 17,949) were in the clopidogrel alone group. Risk of MACE was determined from data from 127,695 patients, MI risk was assessed on the basis of data from 82,330 patients, and risk of CV death was evaluated based on data from 53,905 patients. The PPIs used in the studies were esomeprazole, omeprazole, pantoprazole, rabeprazole, and lansoprazole, but in this meta-analysis as a subgroup analysis we only drew conclusions on the results for omeprazole, esomeprazole, and pantoprazole due to the low number of studies separating data for different PPIs.

### Major adverse cardiac event

**Figure 2:**
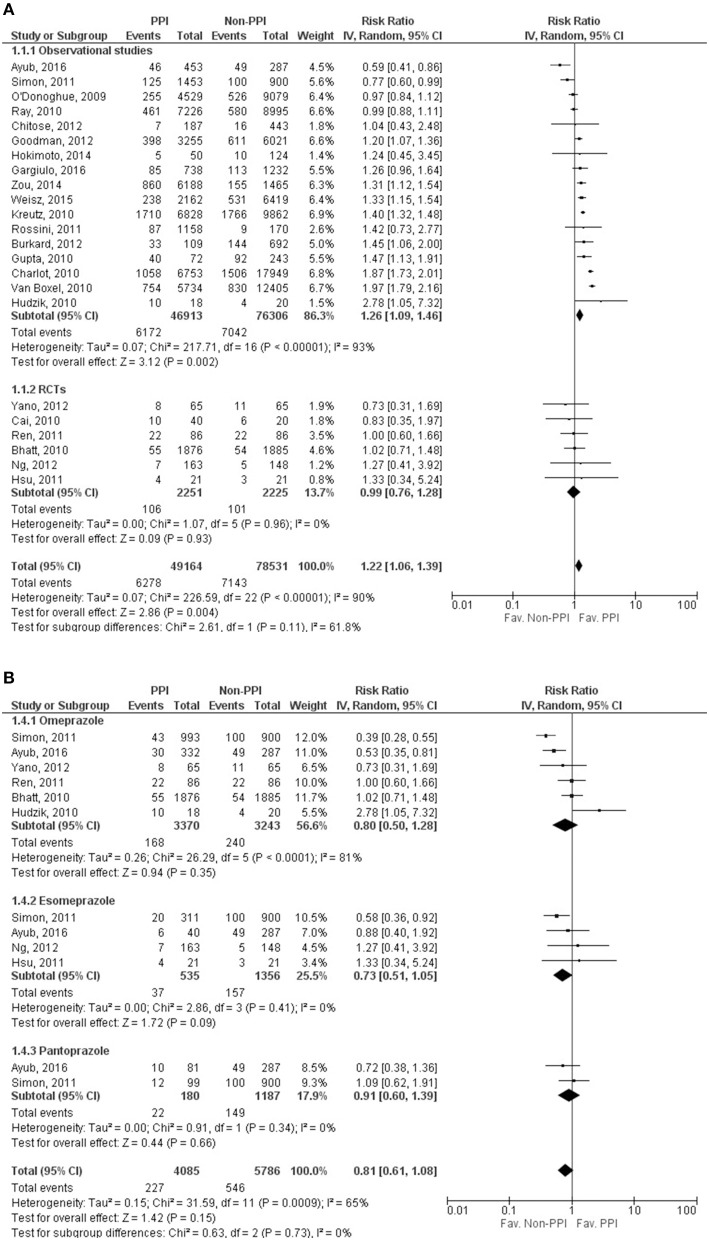
Forrest plots representing the estimated risk of overall major adverse cardiac events **(A)** and in case of taking specific proton pump inhibitors **(B)** CI, confidence interval; PPI, proton pump inhibitor; RCT, randomized controlled trials.

Twenty-three studies (O'Donoghue et al., 2009; Bhatt et al., 2010; Cai et al., 2010; Charlot et al., 2010; Gupta et al., 2010; Hudzik et al., 2010; Kreutz et al., 2010; Ray et al., 2010; van Boxel et al., 2010; Hsu et al., 2011; Ren et al., 2011; Rossini et al., 2011; Simon et al., 2011; Burkard et al., 2012; Chitose et al., 2012; Goodman et al., 2012; Ng et al., 2012; Yano et al., 2012; Hokimoto et al., 2014; Zou et al., 2014; Weisz et al., 2015; Ayub et al., 2016; Gargiulo et al., 2016) reported the incidence of MACE. Our results showed that the risk of MACE is significantly higher in the PPI group (RR = 1.22, 95% CI = 1.06–1.39, p = 0.004), with considerable heterogeneity across the studies included (*I*^2^ = 90%, *p* < 0.001). However, separating the data for the RCT studies from that of the non-RCT studies revealed that a significant association of adverse outcomes (MACE) can only be seen in non-randomized studies (observational studies: RR = 1.26, 95% CI = 1.09–1.46, *p* = 0.002, *I*^2^ = 93%, *p* < 0.001; RCTs: RR = 0.99, 95% CI = 0.76–1.28; *I*^2^ = 0%, *p* = 0.93), although the heterogeneity remained considerable in the observational group, which might not be relevant in the RCT group (Figure 2A, Supplementary Figure 1A). As the result of meta-regression analyses, MACE was not depending on the length of follow up (SE = 0.007, 95% CI = −0.014 to 0.014, *p* = 0.97), based on the results of 18 studies (Bhatt et al., 2010; Charlot et al., 2010; Gupta et al., 2010; Hudzik et al., 2010; Ray et al., 2010; van Boxel et al., 2010; Hsu et al., 2011; Simon et al., 2011; Burkard et al., 2012; Chitose et al., 2012; Goodman et al., 2012; Ng et al., 2012; Yano et al., 2012; Hokimoto et al., 2014; Zou et al., 2014; Weisz et al., 2015; Ayub et al., 2016; Gargiulo et al., 2016), and the age of the patients did not influence the occurrence of the outcome either (SE = 0.023, 95% CI = −0.011 to 0.081, *p* = 0.14), based on the data found in 19 studies (O'Donoghue et al., 2009; Bhatt et al., 2010; Charlot et al., 2010; Gupta et al., 2010; Hudzik et al., 2010; Ray et al., 2010; van Boxel et al., 2010; Hsu et al., 2011; Simon et al., 2011; Burkard et al., 2012; Chitose et al., 2012; Goodman et al., 2012; Ng et al., 2012; Yano et al., 2012; Hokimoto et al., 2014; Zou et al., 2014; Weisz et al., 2015; Ayub et al., 2016; Gargiulo et al., 2016).

In case of patients on omeprazole among the 6 publications included (Bhatt et al., 2010; Hudzik et al., 2010; Ren et al., 2011; Simon et al., 2011; Yano et al., 2012; Ayub et al., 2016), there was no significant difference between the clopidogrel plus PPI and clopidogrel alone groups (RR = 0.80, 95% CI = 0.50–1.28, *p* = 0.35), but since there was evidence of considerable heterogeneity (*I*^2^ = 81%, *p* < 0.001), the random effect model was used for comparison (Figure 2B, Supplementary Figure 1B). In the case of esomeprazole (4 publications, Hsu et al., 2011; Simon et al., 2011; Ng et al., 2012; Ayub et al., 2016), results showed no significant difference in the occurrence of MACE between the groups (RR = 0.73, 95% CI = 0.51–1.05, *p* = 0.09) (Figure 2B, Supplementary Figure 1B). The heterogeneity might not be important (*I*^2^ = 0%, *p* = 0.41); the fixed effects model was used for comparison. In the pantoprazole group, we only found two eligible publications (Simon et al., 2011; Ayub et al., 2016) for MACE, and there was no difference between the two groups (RR = 0.91, 95% CI = 0.60–1.39, *p* = 0.66) (Figure 2B, Supplementary Figure 1B). The heterogeneity might not be important (*I*^2^ = 0%, *p* = 0.34); the fixed effects model was used in analyzing of this specific PPI. The results of analyzing the adjusted events for the overall outcome and for different PPIs are presented as Supplementary Material.

### Cardiovascular death

**Figure 3:**
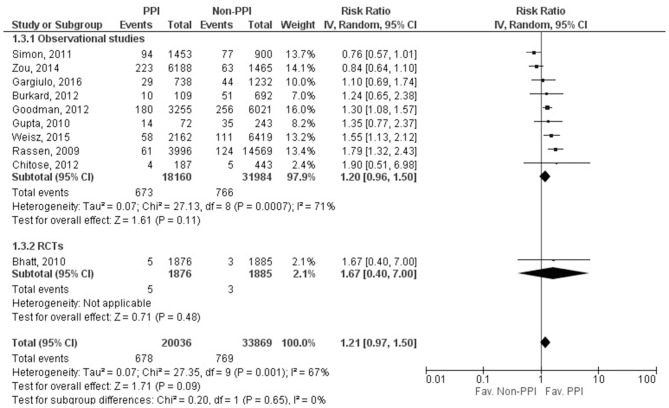
Forrest plot representing the estimated risk of cardiovascular death. CI, confidence interval; PPI, proton pump inhibitor; RCT, randomized controlled trials.

Data on CV death was reported in 10 studies (Rassen et al., 2009; Bhatt et al., 2010; Gupta et al., 2010; Simon et al., 2011; Burkard et al., 2012; Chitose et al., 2012; Goodman et al., 2012; Zou et al., 2014; Weisz et al., 2015; Gargiulo et al., 2016), including 53,905 patients; only one study's data was evaluated as RCT (Bhatt et al., 2010). There was no significant effect of concomitant clopidogrel and PPI treatment on CV death (RR = 1.21, 95% CI = 0.97–1.50, *p* = 0.09). The result from the statistical analysis may represent substantial heterogeneity across the studies (*I*^2^ = 67%, *p* = 0.001). The length of follow up and the age of the patients did not affect the risk for CV death based on results of the included 10 studies (follow up: SE = 0.009, 95% CI = −0.016 to 0.021, *p* = 0.81; age: SE = 0.022; 95% CI = −0.009 to 0.079, *p* = 0.12). Unfortunately, the low amount of data prevented us from evaluating the risk of CV death in specific PPIs (Figure 3, Supplementary Figure 2). Analysis of the adjusted events for CV death can be found in the Supplementary Material.

### Myocardial infarction

**Figure 4:**
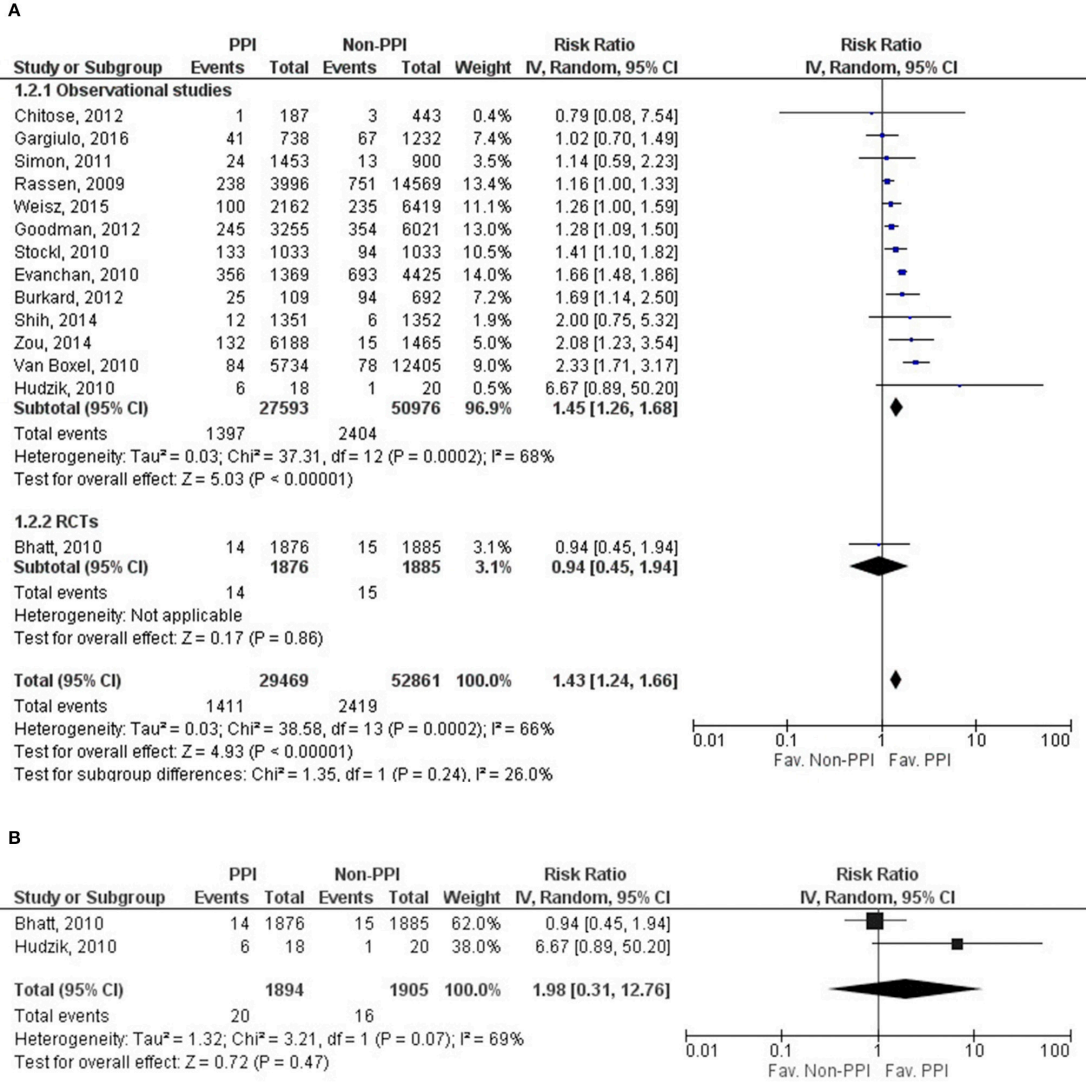
Forrest plots representing the estimated risk of overall myocardial infarction **(A)** and in case of applying omeprazole as proton pump inhibitor **(B)** CI, confidence interval; PPI, proton pump inhibitor; RCT, randomized controlled trials.

Fourteen of the twenty-seven studies contained eligible data on MI, with data for 82,330 patients for evaluation (Rassen et al., 2009; Bhatt et al., 2010; Evanchan et al., 2010; Hudzik et al., 2010; Stockl et al., 2010; van Boxel et al., 2010; Simon et al., 2011; Burkard et al., 2012; Chitose et al., 2012; Goodman et al., 2012; Shih et al., 2014; Zou et al., 2014; Weisz et al., 2015; Gargiulo et al., 2016); one study's data was evaluated as RCT (Bhatt et al., 2010). The risk of MI was significantly higher in the PPI group (RR = 1.43, 95% CI = 1.24–1.66, *p* < 0.001). The results from the statistical analysis may represent substantial heterogeneity across the studies (*I*^2^ = 66%, *p* < 0.001) (Figure 4A, Supplementary Figure 3A). Similarly to MACE and CV death, MI was not depending on the length of follow up or on the patients' age based on the included fourteen studies (follow up: SE = 0.005, 95% CI = −0.005 to 0.013, *p* = 0.41; age: SE = 0.013, 95% CI = −0.045 to 0.007, *p* = 0.15). We only found two eligible articles (Bhatt et al., 2010; Hudzik et al., 2010) for MI in the case of omeprazole, where there was no difference in risk between the observed groups (RR = 1.98, 95% CI = 0.31–12.76, *p* = 0.47). There may be substantial heterogeneity across the studies (*I*^2^ = 69%, *p* = 0.07); the random effects model was used (Figure 4B, Supplementary Figure 3B). We present the result for the analysis of adjusted MI events in the Supplementary Material.

### Risk of bias within studies

Risk of bias was assessed in 17 non-RCT studies (Rassen et al., 2009; Charlot et al., 2010; Evanchan et al., 2010; Gupta et al., 2010; Hudzik et al., 2010; Kreutz et al., 2010; Ray et al., 2010; Stockl et al., 2010; van Boxel et al., 2010; Rossini et al., 2011; Simon et al., 2011; Chitose et al., 2012; Hokimoto et al., 2014; Shih et al., 2014; Zou et al., 2014; Weisz et al., 2015; Ayub et al., 2016), four *post-hoc* analyses of RCTs (O'Donoghue et al., 2009; Burkard et al., 2012; Goodman et al., 2012; Gargiulo et al., 2016), and in six RCTs (Bhatt et al., 2010; Cai et al., 2010; Hsu et al., 2011; Ren et al., 2011; Ng et al., 2012; Yano et al., 2012). The risk of bias within the 27 studies included in this meta-analysis is summarized in the Supplementary Figures 7A,B.

### Publication bias

**Figure 5:**
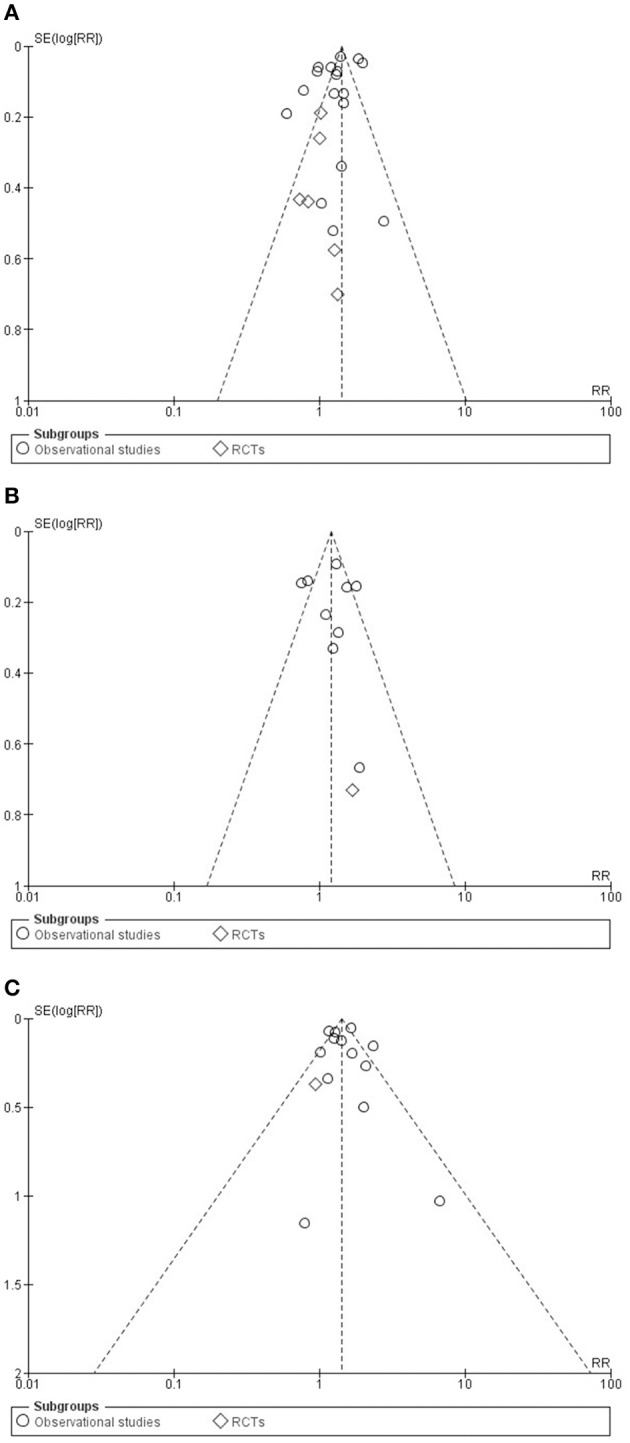
Funnel plots for studies in major adverse cardiac event **(A)**, in cardiovascular death **(B)** and in myocardial infarction **(C)** groups.

Funnel plots were constructed for each outcome and showed symmetry on visual inspection, suggesting that publication bias was not large and was unlikely to alter conclusions (Figures 5A–C).

## Discussion

A possible interaction between clopidogrel and PPIs came to the fore after an observational study had been performed in 2006, which found clopidogrel activity on platelets was diminished in patients receiving PPI treatment (Gilard et al., 2006). Later, this potential interaction was tested in the randomized controlled OCLA (Omeprazole CLopidogrel Aspirin) study, where omeprazole significantly decreased the effect of clopidogrel on *in vitro* platelet activation (Gilard et al., 2008).

Clopidogrel, a thienopyridine derivative, inhibits platelet aggregation through irreversible inhibition of the ADP/P2Y12 receptor on the surface of platelets, and, being a prodrug, it requires a two-step oxidative biotransformation intrahepatically, mediated mainly by cytochrome P450 isoenzymes. First, the cytochrome P450 isoenzymes CYP1A2, CYP2B6, and CYP2C19 form 2-oxo-clopidogrel, which is then oxidized by CYP2B6, CYP2C19, CYP2C9, and CYP3A4 to the active metabolite of clopidogrel, with CYP2C19 being the most important isoenzyme. The active metabolite then binds irreversibly to platelet adenosine diphosphate receptor P2Y12 (Hulot et al., 2006; Disney et al., 2011; Tantry et al., 2011), therefore preventing platelet aggregation. This is associated with the dephosphorylation of the intraplatelet vasodilator-stimulated phosphoprotein. Vasodilator-stimulated phosphoprotein phosphorylation provides an index to evaluate platelet reactivity to clopidogrel (Ward and Kearns, 2013). The findings on mechanisms underlying clopidogrel resistance are contradictory; these mechanisms may relate to heterogeneity in clopidogrel metabolism. CYP2C19 activity can have a profound effect on the conversion of clopidogrel to its active metabolite (Hulot et al., 2006).

All PPIs are extensively metabolized to inactive metabolites mainly via CYP2C19 and CYP3A4 in the liver. Rabeprazole uses these enzymes the least, being mostly converted to its thioether analog non-enzymatically. The potency and specificity of five individual PPIs (omeprazole, esomeprazole, pantoprazole, lansoprazole, rabeprazole) with regard to their inhibitory effects on the activities of four major human CYP enzymes (CYP2C9, CYP2C19, CYP2D6, and CYP3A4) have been studied by Li et al (Li et al., 2004). Lansoprazole was the most potent inhibitor of CYP2C19 enzyme *in vitro*, followed by omeprazole and esomeprazole. Pantoprazole showed the lowest potential to CYP2C19, however it was at least twice as potent an inhibitor as other PPIs toward CYP2C9 and CYP3A4. As the metabolite of rabeprazole, rabeprazole thioether was a strong and competitive inhibitor of CYP2C9, CYP2C19, and CYP2D6. It has been suggested that rabeprazole has significantly less drug-drug interactions than other PPIs, and the main reason is claimed to be its non-enzyme catalyzed degradation, but the results of Li et al suggest that omeprazole and rabeprazole have similar affinity to CYP3A4 (Li et al., 2004; Ogawa and Echizen, 2010). The potential interaction mechanism lies in the fact that both clopidogrel and PPIs, in varying degrees, are metabolized by the same cytochrome P450 enzyme (CYP2C19). PPIs have the potential to competitively inhibit the metabolism of clopidogrel to its active metabolite, which leads to reduced circulating concentrations of the active compound (Disney et al., 2011).

The data on the interactions between clopidogrel and PPIs remain unclear despite the numerous *in vitro* and *in vivo* studies on the subject. The *in vitro* studies have shown that the effectiveness of clopidogrel decreases with simultaneous use of clopidogrel and PPIs (Gilard et al., 2008), and, therefore, the risk for CV events will be elevated. Several possible causative factors may lie behind this phenomenon. One of them is the connected bio-transformational route of clopidogrel and PPIs, or the possible differences in genetic polymorphism of these enzymes (Hulot et al., 2006). There are several studies, mostly observational ones, whose findings are consistent with these *in vitro* results, showing an elevated risk for CV side-effects in patients on combined clopidogrel and PPI treatment (Pezalla et al., 2008; Ho et al., 2009; Juurlink et al., 2009; Kreutz et al., 2010). However, it should be noted that prophylactic PPIs are more likely prescribed to patients with a higher risk for CV events (Disney et al., 2011).

There is considerable disagreement between the various clinical studies that show no increased risk of CV outcomes (O'Donoghue et al., 2009; Rassen et al., 2009; Bhatt et al., 2010; Ray et al., 2010; Zairis et al., 2010). Furthermore, a few studies found no difference in the possible disadvantageous effect of PPI drugs causing extended inhibition of CYP2C19 (O'Donoghue et al., 2009; Zairis et al., 2010). In several cases, the authors used multivariable adjustments for covariates to standardize because the effect of possible factors (such as age, co-morbidities, and co-medication) could modify the outcomes (Rassen et al., 2009; Valkhoff et al., 2011). In a well-designed case-control study, a current PPI plus clopidogrel group result was compared to the results for patients on current clopidogrel plus past PPI therapy. The association between PPI therapy and the recurrence of MI has disappeared suggesting that the appearance of recurrent MI is a result of a residual confounding (Valkhoff et al., 2011).

Based on the ACCF/ACG/AHA 2010 Expert Consensus Document (Abraham et al., 2010) to reduce the risk of GI bleeding, PPIs are recommended among patients with history of upper GI bleeding or with multiple risk factors (e.g., advanced age, concomitant use of warfarin, steroid or NSAIDs, or *H. pylori* infection) for GI bleeding who require antiplatelet therapy. Patients with acute coronary syndrome and prior upper GI bleeding are at substantial CV risk, so dual antiplatelet therapy with concomitant use of a PPI may provide the optimal balance of risk and benefit. The risk reduction achieved by concomitant PPIs might outweigh any potential reduction in the CV efficacy of antiplatelet treatment because of a drug–drug interaction. Routine use of acid suppressant drugs is not recommended for patients at lower risk of upper GI bleeding, who have much less potential to benefit from prophylactic therapy. Clinical decisions regarding concomitant use of PPIs and thienopyridines must be based on whether the potential for benefit outweighs the potential for harm, considering both CV and GI complications. Furthermore, according to the European Cardiology Society's 2017 guideline (Ibanez et al., 2018) for the management of acute myocardial infarction in patients presenting with ST-segment elevation a PPI in combination with dual antiplatelet therapy is recommended (I/B recommendation) in patients at high risk of GI bleeding. Based on the recent European Society of Cardiology/European Association for Cardio-Thoracic Surgery guidelines (Neumann et al., 2018) on myocardial revascularization every effort should be undertaken such as routine use of PPIs to avoid bleeding in patients after percutaneous coronary intervention requiring oral anticoagulation and dual antiplatelet therapy. These statements have been supported by several studies which showed that the risk of upper GI bleeding can be reduced in patients with clopigodrel by concomitant PPI treatment. The occurrence of GI bleeding were 0.2–1.2% (Bhatt et al., 2010), 0–2% (Chitose et al., 2012), 0.4–1.8% (Mo et al., 2015) in the PPI vs. non-PPI groups, respectively.

In this meta-analysis, our aim was to focus on this discrepancy and to find a possible resolution. Our combined data from all of the studies involved showed that the presence of MACE and MI is significantly higher in the PPI plus clopidogrel patient population, a finding which is consistent with results from previous observational studies (Ho et al., 2009; Juurlink et al., 2009; Charlot et al., 2010; Gupta et al., 2010; Hudzik et al., 2010; van Boxel et al., 2010). However, in reducing the degree of heterogeneity by creating subgroups based on study design, we also found that this previously experienced risk elevation and heterogeneity will disappear as in other studies (Kwok and Loke, 2010). This result is similar to those of previous meta-analyses, where a higher CV risk was found among observational studies without any difference between the clopidogrel plus PPI group and the no PPI group in RCTs (Chen et al., 2012). In previous meta-analyses by Mo et al. (2015) and Chen et al. (2013), data only collected from RCTs showed no correlation between simultaneous clopidogrel and PPI therapy and elevated CV risk. An examination of the results, heterogeneity and risk of bias of the studies involved in our meta-analysis points to the low quality of observational studies, whose results are opposite to those of RCT studies, all proving an acceptance of results from RCT studies showing no enhancement of CV risks due to PPIs.

Although our meta-analysis has shown that there is no association between CV risk elevation and PPI usage, our analysis might have limitations. One is that in the 22 studies included, the population had previously had CV diseases, had already undergone percutaneous coronary intervention, or had received dual antiplatelet therapy, meaning that the population under examination may have had severe conditions. In this meta-analysis, we did not analyze the effect of these or other co-morbidities nor evaluate their conditions, but it is possible that the harmful effect of PPIs may be different in patients who need primary or secondary CV prevention. Although we performed secondary analyses on adjusted events, the conclusions drawn from these analyses are limited, because of the insufficient availability of these values across all studies, which were all observational ones, and the applied covariates were different among them. The studies published and available in the databases provided poor descriptions of other risk factors (such as co-morbidities, co-medications, smoking, obesity etc.), preventing us from providing a summary or conclusion in that regard. The other limitation of our study is the substantial heterogeneity among the studies, which may stem from several factors, such as differences in study design. In observational studies or in *post-hoc* analyses of RCTs, the groups were not allocated randomly. It was usually the physicians' decision, so this most likely led to a distortion of the results. Therefore, risk of bias within studies should be highlighted, as well. Though the open-label design might have a less prominent effect on hard CV outcomes, lack of blinding should be mentioned, even in RCTs. In addition, incomplete follow-up and not carefully applied objective evaluation of ascertainment of drug exposures may impose additional risk of bias. Bias is inherent in observational studies, the subgroup analysis of RCTs and observational studies yielding discrepant results support this statement. And there is a problem with the definition of MACE, which is not standard in the literature, although it is most often used to express the CV risk of PPIs plus clopidogrel.

Our aim was to draw conclusion from data for a large patient population; we therefore included as many observational studies as the inclusion criteria permitted despite their limitations. Patients were selected from various ethnic groups; they thus represent the world population. With a few years having passed since previous meta-analyses were published on the subject (the last study in these meta-analyses having been published in 2014) (Mo et al., 2015; Sherwood et al., 2015; Serbin et al., 2016), and with new studies having been carried out since then, we were prompted to perform this systematic search and meta-analysis to re-evaluate the risks.

## Conclusion

Our meta-analysis has shown that there is no definitive evidence for any significant association between CV risk elevation and PPI in patients on clopidogrel treatment, based on RCTs. Thus, no definitive evidence exists for an effect on mortality. From this point of view, the previous FDA guidance to use favorable or non-favorable drug combinations does not seem to be relevant by now based on both previous trials (e.g., COGENT, TRITON-TIMI) and our own analyses. However, taking into account the bias, this meta-analysis should be interpreted with caution, and conducting further RCTs would be beneficial. Because PPI induced risk reduction clearly outweighs the possible adverse CV risk in patients with a high risk of GI bleeding, a combination of clopidogrel with PPI should be recommended.

## Author contributions

All the authors were involved in the study design and edited, read, and approved the final manuscript. During the study, AD and EB performed the literature search and extracted data from the studies involved. KM, AM, ZS, and DP rechecked the studies involved for inclusion and exclusion criteria. PeH, JB, and ÁV formed a committee to decide on points of contention. AD, LC, HA, and ZG assessed the risks of bias in the studies involved. AD and PéH created the risk-related figures. TL performed the statistical analysis and created the forest and funnel plot figures. AD, IS, and PéH drafted the manuscript. All the authors approved the final draft. PéH and IS contributed equally to this article.

### Conflict of interest statement

The authors declare that the research was conducted in the absence of any commercial or financial relationships that could be construed as a potential conflict of interest.

## Abbreviations

CV: cardiovascular
GI: gastrointestinal
MACE: major adverse cardiac event
MI: myocardial infarction
PPI: proton pump inhibitor
RCT: randomized controlled trial
RR: risk ratio/relative risk
