# Supplementary material for: PPIs Are Not Responsible for Elevating Cardiovascular Risk in Patients on Clopidogrel—A Systematic Review and Meta-Analysis
Source: Front Physiol. 2018 Nov 19;9:1550. doi: 10.3389/fphys.2018.01550 (PMC6252380; doi:10.3389/fphys.2018.01550)
Supplement: Supplementary file 1 [file Presentation_1.pdf]

frontiers

SUPPLEMENTARY MATERIAL

Supplementary Table 1A **Baseline characteristics: population**

			Non PPI	PPI	Non PPI	PPI	Non PPI	PPI
**Study**	**Country/region**	**Mean follow-up**	**Male (n)**	**Male (n)**	**Mean age (years)**	**Mean age (years)**	**Mean BMI (kg/m^2)**	**Mean BMI (kg/m^2)**
**Ayub et al, 2016**	South Asia	24 months	216	331	56,2	57,8	26,5	26,3
**Gargiulo et al, 2016**	Italy	24 months	976	535	68,1	71,2	26,9	26,2
**Weisz et al, 2015**	USA, Germany	24 months	4834	1522	63,3	64,6	29,5	29,4
**Hokimoto et al, 2014**	Japan	18 months	89	28	68,8	69,7	24,3	23,3
**Shih et al, 2014**	Taiwan	4 months	64675	64675	49,3	49,3		
**Zou et al, 2014**	China	12 months	1083	4548	65,7	66,2	25,2	25,1
**Burkard et al, 2012**	Switzerland	36 months	75	553	63,3	66,5		
**Chitose et al, 2012**	Japan	18 months	326	139	69,6	69,7	24	24,2
**Goodman et al, 2012**	Europe, Middle East, Africa, Asia, Australia, North America, Central America, South America	12 months	8585	4734	62	63		
**Ng et al, 2012**	Hong Kong	4 months	107	126	63,1	64,3		
**Yano et al, 2012**	Japan	12 months	52	50	66	67		
**Hsu et al, 2011**	Taiwan	6 months	59	65	73,3	70,6		
**Ren et al, 2011**	China	1 month						
**Rossini et al, 2011**	NS	12 months						
**Simon et al, 2011**	France	12 months	644	1058	65	64	27,5	27,1
**Bhatt et al, 2010**	15 countries (NS)	3.5 months	1308	1255	68,7	68,5	28,3	28,4
**Cai et al, 2010**	NS	1 month						
**Charlot et al, 2010**	Denmark	At least 30 days	12801	1775	64,1	67,5		
**Evanchan et al, 2010**	NS	12 months			62,9	63,5		
**Gupta et al, 2010**	USA	50 months			62	61,7		
**Hudzik et al, 2010**	Poland	12 months	13	15	60,5	62,8	27,5	27,1
**Kreutz et al, 2010**	USA	NS						
**Ray et al, 2010**	USA	At least 12 months	4776	3295	60,4	60,8		
**Stockl et al, 2010**	USA	12 months	573	588	68,9	69,2		
**Van Boxel et al, 2010**	Netherlands	At least 12 months	8296	3356	66,1	68,6		
**O'Donoghue et al, 2009**	NS	NS	58	19	64,1	63,1	29	30,6
**Rassen et al, 2009**	USA	6 months	7523	1208	76,8	77,6		

BMI: body mass index; n: number of patients; NS: not specified; PPI: proton pump inhibitor

Supplementary Table 1B **Baseline characteristics: other medications**

	Non PPI	PPI	Non PPI	PPI
**Study**	**ACE-I/ARB (n)**	**ACE-I/ARB (n)**	**Statin (n)**	**Statin (n)**
**Ayub et al, 2016**				
**Gargiulo et al, 2016**			1093	671
**Weisz et al, 2015**				
**Hokimoto et al, 2014**	100	36	118	47
**Shih et al, 2014**	15412	15413	7241	7242
**Zou et al, 2014**	627	2364	1373	5724
**Burkard et al, 2012**				
**Chitose et al, 2012**	148	318	317	124
**Goodman et al, 2012**				
**Ng et al, 2012**				
**Yano et al, 2012**				
**Hsu et al, 2011**				
**Ren et al, 2011**				
**Rossini et al, 2011**				
**Simon et al, 2011**	156	184	220	281
**Bhatt et al, 2010**			1254	1274
**Cai et al, 2010**				
**Charlot et al, 2010**	9129	3708	16002	5684
**Evanchan et al, 2010**	2884	960	3322	1060
**Gupta et al, 2010**	110	39	159	49
**Hudzik et al, 2010**	15	17	18	16
**Kreutz et al, 2010**				
**Ray et al, 2010**				
**Stockl et al, 2010**				
**Van Boxel et al, 2010**	7686	3798	10578	4886
**O'Donoghue et al, 2009**			65	24
**Rassen et al, 2009**	5807	1686	6275	1639

ACE-I: angiotensin-converting enzyme inhibitor; ARB: angiotensin receptor blocker; n: number of patients; PPI: proton pump inhibitor

Supplementary Table 1C **Baseline characteristics: cardio- and cerebrovascular history**

	Non PPI	PPI	Non PPI	PPI	Non PPI	PPI
**Study**	**PCI (n)**	**PCI (n)**	**MI (n)**	**MI (n)**	**Stroke (n)**	**Stroke (n)**
**Ayub et al, 2016**						
**Gargiulo et al, 2016**	229	119	321	199		
**Weisz et al, 2015**	2651	1025	1547	618		
**Hokimoto et al, 2014**			35	9	22	5
**Shih et al, 2014**					15610	15609
**Zou et al, 2014**			290	1071		
**Burkard et al, 2012**	115	15	24	193		
**Chitose et al, 2012**			100	55	45	29
**Goodman et al, 2012**						
**Ng et al, 2012**						
**Yano et al, 2012**	1,3	5	0	4		
**Hsu et al, 2011**			54	59	27	33
**Ren et al, 2011**						
**Rossini et al, 2011**						
**Simon et al, 2011**	93	96	125	141	35	41
**Bhatt et al, 2010**	1334	1331	566	531	136	151
**Cai et al, 2010**						
**Charlot et al, 2010**						
**Evanchan et al, 2010**						
**Gupta et al, 2010**						
**Hudzik et al, 2010**			14	13		
**Kreutz et al, 2010**						
**Ray et al, 2010**					1700	1503
**Stockl et al, 2010**	827	825	546	554	8	4
**Van Boxel et al, 2010**			4163	2001	370	203
**O'Donoghue et al, 2009**			17	11		
**Rassen et al, 2009**			954	223		

MI: myocardial infarction; PCI: percutaneous coronary intervention; n: number of patients; PPI: proton pump inhibitor

Supplementary Table 1D **Baseline characteristics: cardiovascular risk factors**

Study	Non PPI Hypertension (n)	PPI Hypertension (n)	Non PPI DM (n)	PPI DM (n)	Non PPI Dyslipidaemia (n)	PPI Dyslipidaemia (n)	Non PPI Smoking (n)	PPI Smoking (n)
**Ayub et al, 2016**	162	294	106	177	131	176		
**Gargiulo et al, 2016**	879	535	305	172	681	397	301	167
**Weisz et al, 2015**	5039	1790	2080	703	4731	1645	1464	480
**Hokimoto et al, 2014**	97	36	57	18	85	32	20	9
**Shih et al, 2014**	43420	43420	27229	27230	38105	38105		
**Zou et al, 2014**	1031	4412	346	1597	913	1597	454	1993
**Burkard et al, 2012**	450	79	119	32	80	525	206	27
**Chitose et al, 2012**	349	144	151	64	257	110	113	48
**Goodman et al, 2012**								
**Ng et al, 2012**							28	32
**Yano et al, 2012**	44	44	10	19	40	39	37	40
**Hsu et al, 2011**	57	56	23	35			5	10
**Ren et al, 2011**								
**Rossini et al, 2011**								
**Simon et al, 2011**	481	749	314	433	431	614	301	512
**Bhatt et al, 2010**	1497	1526	593	536	1478	1446	234	265
**Cai et al, 2010**								
**Charlot et al, 2010**								
**Evanchan et al, 2010**	2835	837	1601	630	2734	850		
**Gupta et al, 2010**	166	55	73	26	146	48	81	18
**Hudzik et al, 2010**	14	13	6	8	15	13		
**Kreutz et al, 2010**								
**Ray et al, 2010**								
**Stockl et al, 2010**	5	10	3	6				
**Van Boxel et al, 2010**								
**O'Donoghue et al, 2009**	55	22	18	11	61	25	7	9
**Rassen et al, 2009**	9577	2894	4619	1389				

DM: diabetes mellitus; n: number of patients; PPI: proton pump inhibitor

SUPPLEMENTARY RESULTS

Results of fixed effects models

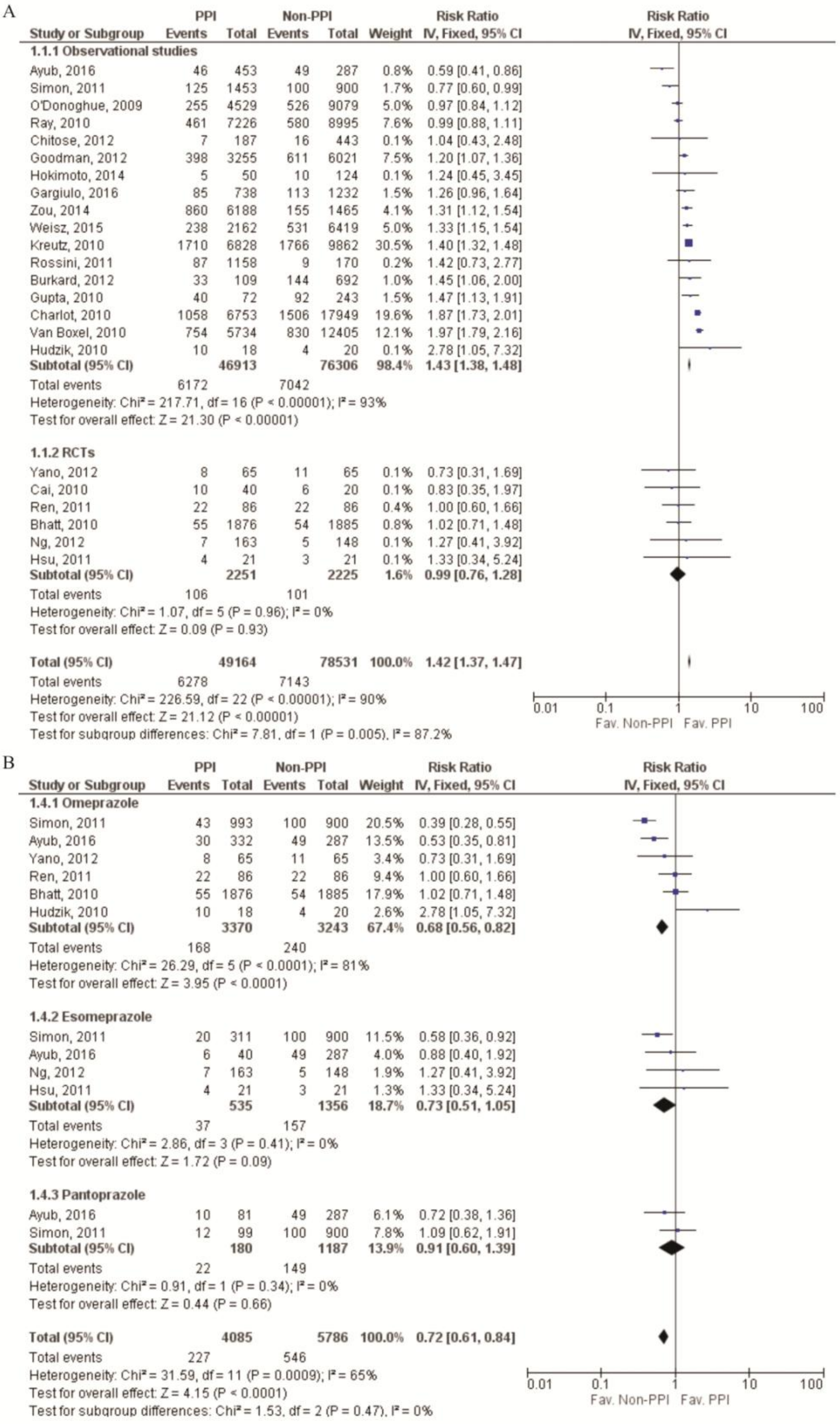

Supplementary Figure 1 Forrest plots representing the estimated risk of major adverse cardiac event using fixed effects model. (A: overall outcome; B: outcome occurrence in case of different PPIs) *CI: confidence interval; PPI: proton pump inhibitor; RCT: randomized controlled trials.*

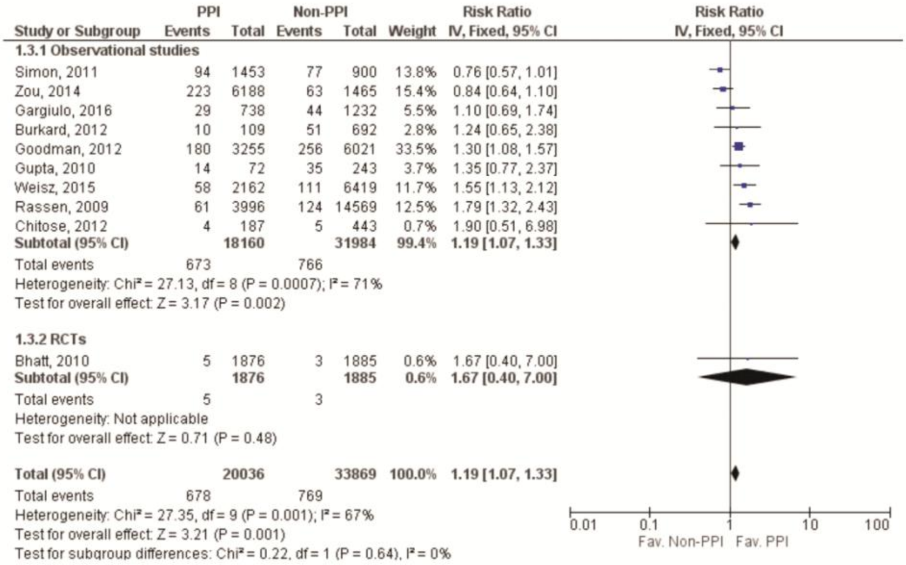

Supplementary Figure 2 Forrest plot representing the estimated risk of cardiovascular death using fixed effects model. *CI: confidence interval; PPI: proton pump inhibitor; RCT: randomized controlled trials.*

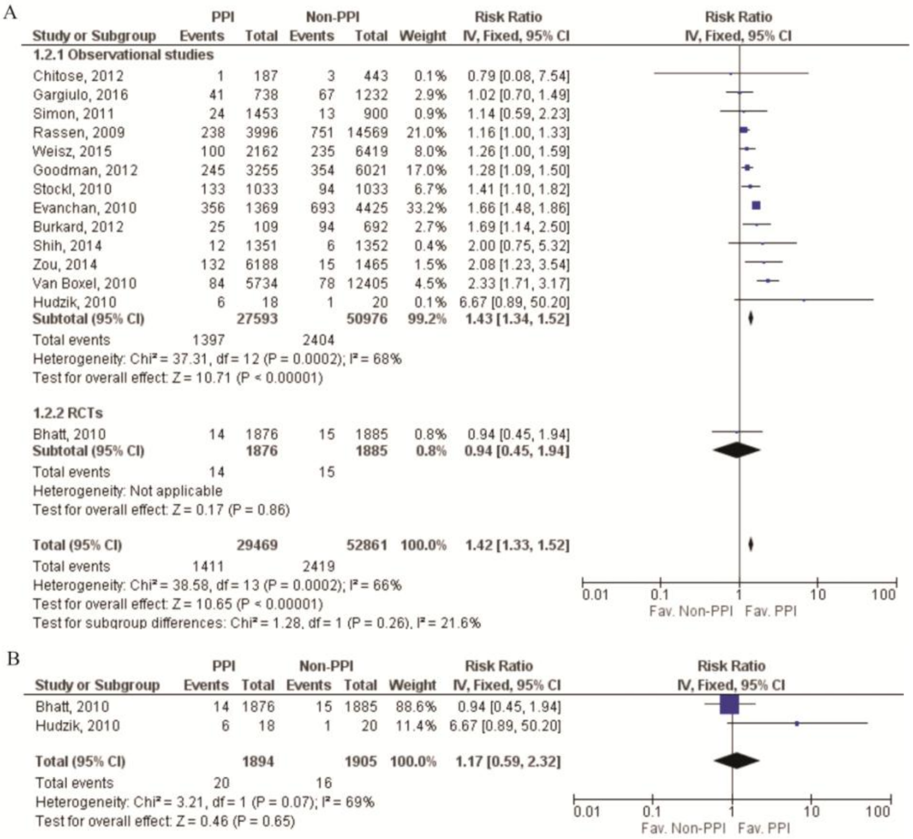

Supplementary Figure 3 Forrest plots representing the estimated risk of myocardial infarction using fixed effects model. (A: overall outcome; B: outcome occurrence in case of omeprazole) *CI: confidence interval; PPI: proton pump inhibitor; RCT: randomized controlled trials.*

Statistical analysis of adjusted hazard ratios

Adjusted hazard ratios (HRs) for all three major outcomes (major adverse cardiac event (MACE), cardiovascular (CV) death, myocardial infarction (MI) were available from observational studies.

Six studies reported adjusted HRs for overall MACE outcome (Ray et al., 2010; van Boxel et al., 2010; Goodman et al., 2012; Zou et al., 2014; Weisz et al., 2015; Gargiulo et al., 2016). The results showed that the risk of MACE was significantly higher in the clopidogrel plus PPI group (HR=1.25, 95% CI=1.03–1.51, p=0.02) (**Supplementary Figure 4A–B**). We have found considerable heterogeneity across the included studies (I^2=88%, p<0.001); the random effects model was used. In the case of specific PPIs, four studies (O'Donoghue et al., 2009; Charlot et al., 2010; Kreutz et al., 2010; Ray et al., 2010) presented data on adjusted HRs for omeprazole, esomeprazole and pantoprazole (**Supplementary Figure 4C–D**). The results showed that there is no difference between the clopidogrel alone and clopidogrel plus PPI groups in case of esomeprazole (HR=1.17, 95% CI=0.90–1.53, p=0.25), omeprazole (HR=1.12, 95% CI=0.86–1.45, p=0.41), and pantoprazole (HR=1.25, 95% CI=0.99–1.57, p=0.06). In the specified PPI groups, we also found considerable heterogeneity (esomeprazole: 84%, p<0.001; omeprazole: 82%, p=0.001; pantoprazole: 85%, p<0.001), the random effects model was used for the analysis.

Two studies contained eligible data on CV death (Goodman et al., 2012; Gargiulo et al., 2016) (**Supplementary Figure 5A-B**), and according to their results, there was no significant effect of concomitant clopidogrel and PPI treatment on CV death (HR=1.16, 95% CI=0.72–1.87, p=0.53). Data on MI was reported in six studies (Stockl et al., 2010; van Boxel et al., 2010; Goodman et al., 2012; Shih et al., 2014; Zou et al., 2014; Gargiulo et al., 2016) (**Supplementary Figure 6A–B**), and the risk for MI was significantly higher in the PPI plus clopidgorel group (HR=1.46, 95% CI=1.08–1.96, p=0.01). For the CV death and MI outcomes the heterogeneity may represent substantial-moderate heterogeneity (70–60%, p=0.07–0.03, respectively), the random effects model was used for both outcomes.

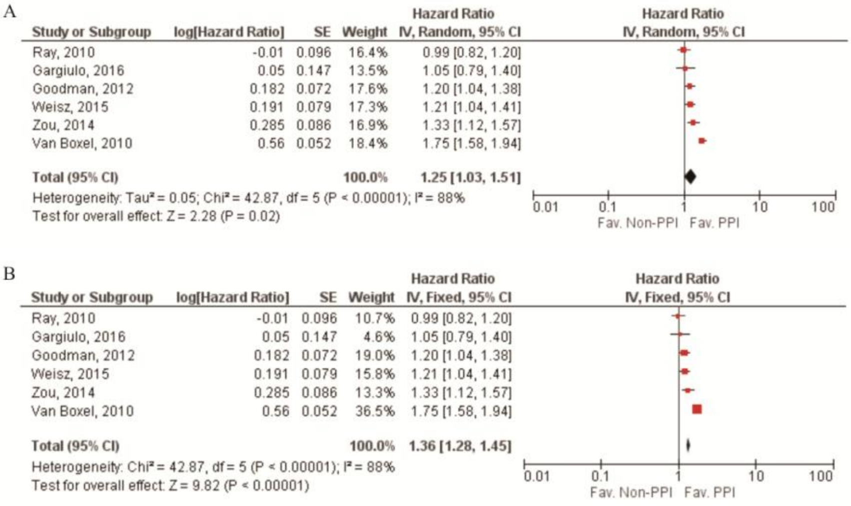

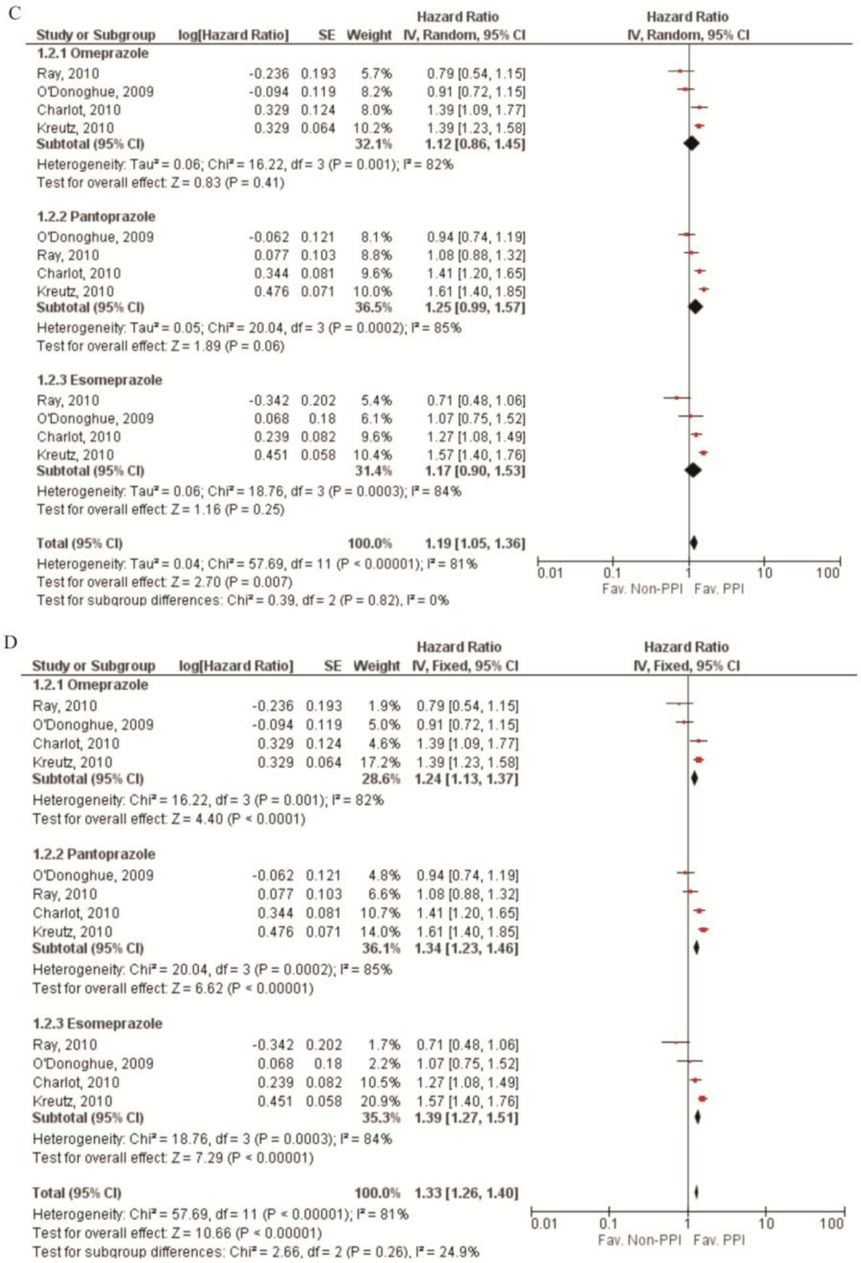

Supplementary Figure 4 Forrest plots representing the analysis of adjusted events for overall major adverse cardiac events (A–B), and for different PPIs (C–D) using random (A, C) and fixed (B, D) effects models.
CI: confidence interval; PPI: proton pump inhibitor.

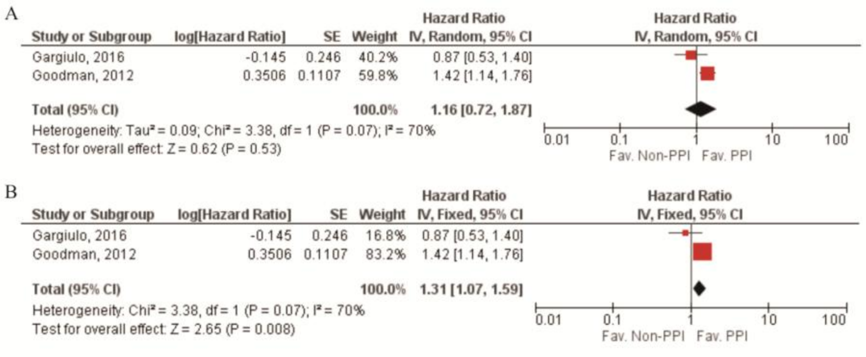

Supplementary Figure 5 Forrest plots representing the analysis of adjusted events for cardiovascular death, using random (A) and fixed (B) effects models. *CI: confidence interval; PPI: proton pump inhibitor.*

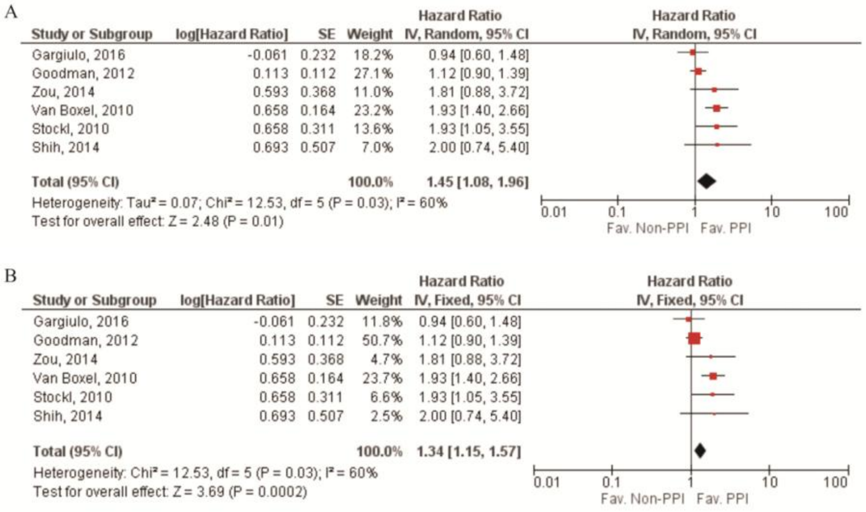

Supplementary Figure 6 Forrest plots representing the analysis of adjusted events for myocardial infarction, using random (A) and fixed (B) effects models. *CI: confidence interval; PPI: proton pump inhibitor.*

Abbreviations

CV: cardiovascular
HR: hazard ratio
MACE: major adverse cardiac event
MI: myocardial infarction

References

Charlot, M., Ahlehoff, O., Norgaard, M.L., Jorgensen, C.H., Sorensen, R., Abildstrom, S.Z., et al. (2010). Proton-pump inhibitors are associated with increased cardiovascular risk independent of clopidogrel use: a nationwide cohort study. *Ann Intern Med* 153(6)**,** 378-386. doi: 10.7326/0003-4819-153-6-201009210-00005.

Gargiulo, G., Costa, F., Ariotti, S., Biscaglia, S., Campo, G., Esposito, G., et al. (2016). Impact of proton pump inhibitors on clinical outcomes in patients treated with a 6- or 24-month dual-antiplatelet therapy duration: Insights from the PROlonging Dual-antiplatelet treatment after Grading stent-induced Intimal hyperplasia studY trial. *Am Heart J* 174**,** 95-102. doi: 10.1016/j.ahj.2016.01.015.

Goodman, S.G., Clare, R., Pieper, K.S., Nicolau, J.C., Storey, R.F., Cantor, W.J., et al. (2012). Association of proton pump inhibitor use on cardiovascular outcomes with clopidogrel and ticagrelor: insights from the platelet inhibition and patient outcomes trial. *Circulation* 125(8)**,** 978-986. doi: 10.1161/circulationaha.111.032912.

Kreutz, R.P., Stanek, E.J., Aubert, R., Yao, J., Breall, J.A., Desta, Z., et al. (2010). Impact of proton pump inhibitors on the effectiveness of clopidogrel after coronary stent placement: the clopidogrel Medco outcomes study. *Pharmacotherapy* 30(8)**,** 787-796. doi: 10.1592/phco.30.8.787.

O'Donoghue, M.L., Braunwald, E., Antman, E.M., Murphy, S.A., Bates, E.R., Rozenman, Y., et al. (2009). Pharmacodynamic effect and clinical efficacy of clopidogrel and prasugrel with or without a proton-pump inhibitor: an analysis of two randomised trials. *Lancet* 374(9694)**,** 989-997. doi: 10.1016/s0140-6736(09)61525-7.

Ray, W.A., Murray, K.T., Griffin, M.R., Chung, C.P., Smalley, W.E., Hall, K., et al. (2010). Outcomes with concurrent use of clopidogrel and proton-pump inhibitors: a cohort study. *Ann Intern Med* 152(6)**,** 337-345. doi: 10.7326/0003-4819-152-6-201003160-00003.

Shih, C.J., Chen, Y.T., Ou, S.M., Li, S.Y., Chen, T.J., and Wang, S.J. (2014). Proton pump inhibitor use represents an independent risk factor for myocardial infarction. *Int J Cardiol* 177(1)**,** 292-297. doi: 10.1016/j.ijcard.2014.09.036.

Stockl, K.M., Le, L., Zakharyan, A., Harada, A.S., Solow, B.K., Addiego, J.E., et al. (2010). Risk of rehospitalization for patients using clopidogrel with a proton pump inhibitor. *Arch Intern Med* 170(8)**,** 704-710. doi: 10.1001/archinternmed.2010.34.

van Boxel, O.S., van Oijen, M.G., Hagenaars, M.P., Smout, A.J., and Siersema, P.D. (2010). Cardiovascular and gastrointestinal outcomes in clopidogrel users on proton pump inhibitors: results of a large Dutch cohort study. *Am J Gastroenterol* 105(11)**,** 2430-2436; quiz 2437. doi: 10.1038/ajg.2010.334.

Weisz, G., Smilowitz, N.R., Kirtane, A.J., Rinaldi, M.J., Parvataneni, R., Xu, K., et al. (2015). Proton Pump Inhibitors, Platelet Reactivity, and Cardiovascular Outcomes After Drug-Eluting Stents in Clopidogrel-Treated Patients: The ADAPT-DES Study. *Circ Cardiovasc Interv* 8(10). doi: 10.1161/circinterventions.114.001952.

Zou, J.J., Chen, S.L., Tan, J., Lin, L., Zhao, Y.Y., Xu, H.M., et al. (2014). Increased risk for developing major adverse cardiovascular events in stented Chinese patients treated with dual antiplatelet therapy after concomitant use of the proton pump inhibitor. *PLoS One* 9(1)**,** e84985. doi: 10.1371/journal.pone.0084985.

RISK OF BIAS ASSESSMENT

Cochrane Risk of Bias Tool

1.) **Study:** Bhatt et al, 2010

A) **Random sequence generation**
Review authors' judgment: Unclear risk
Notes on rating: No information is available about random sequence generation (permuted block randomization with stratification by H. pylori status and concomitant NSAID use).

B) **Allocation concealment**
Review authors' judgment: Low risk
Notes on rating: Randomization was performed centrally by CRO.

C) **Blinding of participants and personnel**
Review authors' judgment: Low risk
Notes on rating: Blinded.

D) **Blinding of outcome assessment**
Review authors' judgment: Low risk.
Notes on rating: Blinded.

E) **Incomplete outcome data**
Review authors' judgment: Low risk.
Notes on rating: Planned drop-out: maximum 20%, 3,761 of 3,873 randomized patients were included in analysis (drop-out: 2.8%). The negligible drop-out is unlikely to introduce bias.

F) **Selective reporting**
Review authors' judgment: Low risk.
Notes on rating: Study protocol is available at nejm.org (detailed) and ClinicalTrials.gov (brief) (NCT00557921), all relevant outcomes are reported.

G) **Other sources of bias**
Review authors' judgment: High risk.
Notes on rating: The study ended prematurely due to lack of financial resources (the planned number of gastrointestinal events was not reached).

2.) **Study:** Hsu et al, 2011

A) **Random sequence generation**
Review authors' judgment: Low risk
Notes on rating: Computer-generated sequence.

B) **Allocation concealment**
Review authors' judgment: Low risk
Notes on rating: Consecutively numbered sealed envelopes.

C) **Blinding of participants and personnel**
Review authors' judgment: High risk.
Notes on rating: Open label.

D) **Blinding of outcome assessment**

Review authors' judgment: High risk.

Notes on rating: Open label.

E) **Incomplete outcome data**

Review authors' judgment: Low risk.

Notes on rating: Balanced drop-out from both study arms (4.5% vs. 5.1%). Both intention-to-treat and per-protocol analyses were performed.

F) **Selective reporting**

Review authors' judgment: Low risk.

Notes on rating: Study protocol is available at ClinicalTrials.gov (NCT01138969), all relevant outcomes are reported.

G) **Other sources of bias**

Review authors' judgment: Unclear risk.

Notes on rating: In intention-to-treat analysis, patients missing final endoscopy were assumed to have normal findings. This assumption may impose risk of bias.

3.) **Study:** Ng et al, 2012

A) **Random sequence generation**

Review authors' judgment: Low risk.

Notes on rating: Randomly shuffled envelopes.

B) **Allocation concealment**

Review authors' judgment: Low risk.

Notes on rating: Identical, blinded, and sealed envelopes.

C) **Blinding of participants and personnel**

Review authors' judgment: Low risk.

Notes on rating: Blinded.

D) **Blinding of outcome assessment**

Review authors' judgment: Low risk.

Notes on rating: Blinded.

E) **Incomplete outcome data**

Review authors' judgment: Low risk.

Notes on rating: One patient has withdrawn before the first dose of the drug from each arm. Drop-out is unlikely to introduce bias.

F) **Selective reporting**

Review authors' judgment: Low risk.

Notes on rating: Study protocol is available at ClinicalTrials.gov (NCT00683111), all relevant outcomes are reported.

G) **Other sources of bias**

Review authors' judgment: Low risk.

Notes on rating: The study is free of other sources of bias.

4.) **Study:** Yano et al, 2012

A) **Random sequence generation**

Review authors' judgment: Low risk

Notes on rating: Computer-generated sequence.

B) **Allocation concealment**

Review authors' judgment: Unclear risk

Notes on rating: Randomization was performed at the central registration site (details about the personnel/party involved in the process are not provided).

C) **Blinding of participants and personnel**

Review authors' judgment: High risk.

Notes on rating: Open label.

D) **Blinding of outcome assessment**

Review authors' judgment: High risk.

Notes on rating: Open label.

E) **Incomplete outcome data**

Review authors' judgment: High risk.

Notes on rating: Although the drop-out is balanced between study-arms and the loss is justified in details, drop-out rate is considerably high (28%), which is likely to introduce bias. The authors did not perform intention-to-treat analysis.

F) **Selective reporting**

Review authors' judgment: Unclear risk.

Notes on rating: We failed to identify protocol of the study, however, the study reported on all outcomes mentioned in the Methods section of the article. Possibility of selective outcome reporting cannot be excluded.

G) **Other sources of bias**

Review authors' judgment: Low risk.

Notes on rating: The study is free of other sources of bias.

Randomized controlled trials	Random sequence generation (Selection bias)	Allocation concealment (Selection bias)	Blinding of participants and personnel (Performance bias)	Blinding of outcome assessment (Detection bias)	Incomplete outcome data (Attrition bias)	Selective reporting (Reporting bias)	Other bias
Ng et al, 2012	+	+	+	+	+	+	+
Yano et al, 2012	+	?	−	−	−	?	+
Hsu et al, 2011	+	+	−	−	+	+	?
Ren et al, 2011	?	?	?	?	+	+	+
Bhatt et al, 2010	?	+	+	+	+	+	−
Cai et al, 2010	+	?	−	?	−	−	?

Supplementary Figure 7A Quality assessment of randomized controlled trials. *Green: low risk of bias; yellow: uncertain risk of bias; red: high risk of bias; BMI: body mass index.*

Modified Newcastle-Ottawa Scale

1. Representativeness of the exposed group:

Low risk: Patients are truly representative to the average population receiving clopidogrel plus proton pump inhibitor.

High risk: Any unjustified inclusion or exclusion criteria applied (e.g., inclusion of patients above 60 years of age exclusively, or exclusion of patients with low cardiovascular risk).

Uncertain risk: No (or unsatisfactory) information about the inclusion or exclusion of patients.

2. Selection of the non-exposed group:

Low risk: Patients are truly representative to the average population receiving clopidogrel without proton pump inhibitor.

High risk: Any unjustified inclusion or exclusion criteria applied (e.g., inclusion of patients above 60 years of age exclusively or exclusion of patients with low cardiovascular risk).

Uncertain risk: No (or unsatisfactory) information about the inclusion or exclusion of patients.

3. Ascertainment of exposure:

Low risk: Objective assessment of compliance regarding proton pump inhibitor intake (e.g., laboratory tests).

High risk: Subjective assessment of complicance regarding PPI intake (e.g., questionnaire).

Uncertain risk: No information.

4. Demonstration that outcome of interest was not present at start of study:

Low risk: All outcomes were not present at the start of the study.

High risk: Any outcome was present at the start of the study.

Uncertain risk: No information.

5A. Study controls for age:

Low risk: No difference in age between groups (statistically verified).

High risk: Difference in age between groups (statistically verified).

Uncertain risk: No comparison made within the study.

5B. Study controls for body mass index:

Low risk: No difference in age between groups (statistically verified).

High risk: Difference in age between groups (statistically verified).

Uncertain risk: No comparison made within the study.

6. Was follow-up long enough for outcomes to occur:

Low risk: At least 1 month follow-up.

High risk: Less than 1 month follow-up.

Uncertain risk: No information about the length of follow-up.

7. Adequacy of follow up of cohorts:

Low risk: Complete follow-up or the drop-out is unlikely to introduce bias (e.g., negligible number of loss, random drop-out).

High risk: Incomplete follow-up which is likely to introduce bias (e.g., non-random drops due to adverse effects).

Uncertain risk: No information about the loss.

Observational studies	Representativeness of the exposed (Selection bias)	Selection of the non-exposed (Selection bias)	Ascertainment of exposure (Selection bias)	Demonstration that outcome of interest was not present at start of study (Selection bias)	Study controls for age (Comparability bias)	Study controls for BMI (Comparability bias)	Was follow-up long enough for outcomes to occur (Outcome bias)	Adequacy of follow-up of cohorts (Outcome bias)
Ayub et al, 2016	+	+	?	?	+	+	+	?
Gargiulo et al, 2016	+	+	?	−	−	+	+	+
Weisz et al, 2015	+	+	?	−	−	+	+	+
Hokimoto et al, 2014	+	+	?	?	+	+	+	?
Shih et al, 2014	+	+	?	?	+	?	+	?
Zou et al, 2014	+	+	−	−	+	+	+	+
Burkard et al, 2012	+	+	?	−	−	?	+	+
Chitose et al, 2012	+	+	?	−	+	+	+	?
Goodman et al, 2012	+	+	−	−	−	?	+	+
Rossini et al, 2011	+	+	?	?	?	?	+	?
Simon et al, 2011	+	+	?	−	−	+	+	?
Charlot et al, 2010	+	+	?	−	−	?	+	+
Evanchan et al, 2010	+	+	−	−	?	?	+	?
Gupta et al, 2010	+	+	?	−	+	?	+	?
Hudzik et al, 2010	+	+	?	−	+	+	+	+
Kreutz et al, 2010	+	+	−	−	−	?	+	?
Ray et al, 2010	+	+	?	−	+	?	+	?
Stockl et al, 2010	+	+	−	−	+	?	+	+
Van Boxel et al, 2010	+	+	−	?	−	?	+	?
Rassen et al, 2009	−	−	−	−	?	?	+	?
O'Donoghue et al, 2009	+	+	?	−	−	−	?	+

Supplementary Figure 7B Modified Newcastle–Ottawa scale for risk of bias assessment of observational studies. *Green: low risk of bias; yellow: uncertain risk of bias; red: high risk of bias; BMI: body mass index.*
